# Structural basis for misregulation of kinesin KIF21A autoinhibition by CFEOM1 disease mutations

**DOI:** 10.1038/srep30668

**Published:** 2016-08-03

**Authors:** Sarah Bianchi, Wilhelmina E. van Riel, Sebastian H. W. Kraatz, Natacha Olieric, Daniel Frey, Eugene A. Katrukha, Rolf Jaussi, John Missimer, Ilya Grigoriev, Vincent Olieric, Roger M. Benoit, Michel O. Steinmetz, Anna Akhmanova, Richard A. Kammerer

**Affiliations:** 1 Laboratory of Biomolecular Research, Division of Biology and Chemistry, Paul Scherrer Institute, CH-5232 Villigen PSI, Switzerland; 2 Cell Biology, Faculty of Science, Utrecht University, 3584 CH, Utrecht, The Netherlands; 3 Swiss Light Source, Paul Scherrer Institute, CH-5232 Villigen PSI, Switzerland

## Abstract

Tight regulation of kinesin activity is crucial and malfunction is linked to neurological diseases. Point mutations in the *KIF21A* gene cause congenital fibrosis of the extraocular muscles type 1 (CFEOM1) by disrupting the autoinhibitory interaction between the motor domain and a regulatory region in the stalk. However, the molecular mechanism underlying the misregulation of KIF21A activity in CFEOM1 is not understood. Here, we show that the KIF21A regulatory domain containing all disease-associated substitutions in the stalk forms an intramolecular antiparallel coiled coil that inhibits the kinesin. CFEOM1 mutations lead to KIF21A hyperactivation by affecting either the structural integrity of the antiparallel coiled coil or the autoinhibitory binding interface, thereby reducing its affinity for the motor domain. Interaction of the KIF21A regulatory domain with the KIF21B motor domain and sequence similarities to KIF7 and KIF27 strongly suggest a conservation of this regulatory mechanism in other kinesin-4 family members.

Kinesin (KIF) motor proteins play a major role in the transport of intracellular cargo and in the regulation of microtubule (MT) organization and dynamics in an ATP-dependent fashion. The kinesin superfamily is divided into 14 families (kinesin-1 to kinesin-14), which are encoded by more than 45 different kinesin genes in the mouse and human genome^1^. A prototypic kinesin includes a conserved globular N-terminal motor domain, which binds to MTs, hydrolyses ATP and converts its chemical energy to mechanical work. The kinesin motor domain is typically followed by a stalk domain often consisting of α-helical coiled-coil regions that are important for dimerization and a tail domain containing the binding sites for cargo or kinesin-regulatory proteins. A tight control of kinesin motor activity is not only crucial for avoiding futile ATP hydrolysis but also for proper motor function. Accordingly, overexpression and misregulation of kinesins is often related to cancer and severe neurodegenerative or neurodevelopmental diseases^2^,^3^.

Up to date, several kinesin regulatory mechanisms have been described^4^,^5^,^6^,^7^. One of these, kinesin autoinhibition, was first reported for KIF5 (kinesin-1) and subsequently identified in KIF17 (kinesin-2), GAKIN/KIF13B, KIF1A/Unc-104 (kinesin-3) and CENP-E (kinesin-7). In the absence of cargo, these motor proteins are inactivated mostly through intramolecular interactions between the motor domain and tail or stalk moieties, which prevent MT binding and/or ATP hydrolysis. Although autoinhibition seems to be a general principle for the regulation of kinesin motors, different autoinhibitory mechanisms were identified. For example, the activity of KIF5 is controlled by two intramolecular autoinhibitory interactions, one affecting ATPase activity and one interfering with MT binding^8^,^9^. It was shown that a tail peptide of the KIF5 heavy chain locks the two motor heads in a rigid conformation, thereby inhibiting ADP release. In addition, the interaction of the motor domain with the KIF5 light chain reduces MT binding^8^,^9^. KIF17 contains a predicted coiled-coil region located in its stalk domain and a tail region that inhibit the motor’s processive motility and MT binding, respectively^10^. In the KIF1A motor, a domain-swapped forkhead-associated domain with an adjacent coiled-coil segment appears responsible for self-regulation, although a direct interaction between the regulatory region and the motor domain could not be demonstrated^11^. Currently, the molecular mechanisms underlying kinesin autoinhibition are only poorly understood, a major reason being the lack of structural information. Only two high-resolution crystal structures of kinesin autoinhibitory domains are available: a KIF1A polypeptide chain fragment containing the coiled-coil segment and the forkhead-associated domain and dimeric KIF5 motor in complex with the inhibitory tail peptide^8^,^11^. The importance of high-resolution structural information is emphasized by the fact that the latter crystal structure could resolve contradictory models on KIF5 autoinhibition.

Two recent studies suggested a self-regulation mechanism for the kinesin-4 family member KIF21A^12^,^13^. KIF21A is a plus-end directed motor, which is most prominently expressed in the central nervous system (CNS)^14^. KIF21A was shown to reduce the MT polymerization rate and inhibit catastrophes *in vitro* and in cells, suggesting a regulatory role in MT dynamics^12^,^13^. Increased KIF21A expression correlates with a change in axon morphology, resulting in axon guidance abnormalities^13^. The importance of KIF21A in the CNS is further demonstrated by the observation that several point mutations in the *KIF21A* gene cause CFEOM1. This neurological disease is characterized by an abnormal development of the oculomotor nerve, resulting in the defective innervation and dysfunction of the muscles elevating the eye lid and eye globe^15^ (reviewed in ref. 16). Notably, all CFEOM1 mutations and a one-residue deletion cluster within a predicted coiled-coil region of the KIF21A stalk or within the motor domain (Fig. 1)^17^. It was shown that a part of the KIF21A stalk containing the residues involved in CFEOM1 (*hs*KIF21A aa701-1180, *mm*KIF21A aa891-1300) binds to its own motor domain, thereby inhibiting the kinesin^12^,^13^. By introducing into the same stalk construct the most common CFEOM1 mutation, R954W, the intra-molecular interaction was disrupted, suggesting that CFEOM1 is caused by a hyperactive KIF21A motor. Both homozygous and heterozygous knock-in mice harboring the R943W (equivalent to human R954W) mutation in the *Kif21a* gene recapitulated the human CFEOM1 pathology, confirming developmental oculomotor axon stalling as a major disease cause^12^.

Although the biological mechanism underlying CFEOM1 in the neuronal environment has been clarified by work, detailed molecular information underlying KIF21A autoinhibition is still missing. To address this important issue, we characterized in detail the interaction between the KIF21A motor domain and its regulatory stalk region (Fig. 1) using a multidisciplinary approach, including biophysical, structural biology and cell biological methods.

## Results

### The KIF21A stalk peptide rCC1 forms an intramolecular antiparallel coiled coil in solution

**Figure 1:**
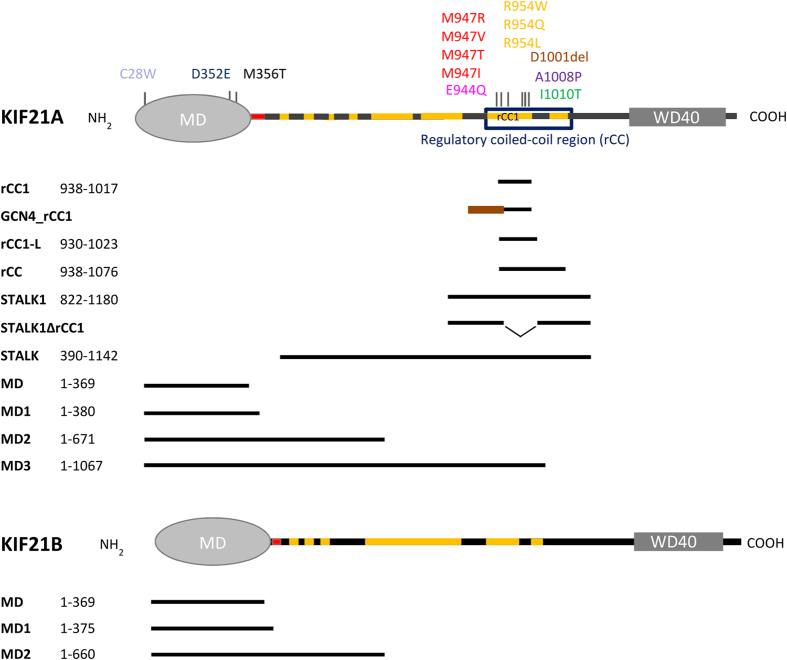
Schematic representation of all constructs used in this study. Schematic representation of the KIF21A (UniProt: Q7Z4S6) and KIF21B (UniProt: O75037) motors. Domains are indicated. Predicted coiled-coil and non-coiled-coil regions in the stalk are shown in orange and black, respectively. The regulatory coiled-coil region (rCC) and the neck region are highlighted by a box and in red, respectively. CFEOM1 mutations are indicated in different colors. Fragments are represented by black lines and the GCN4 coiled coil is shown in brown.

Towards understanding the mechanism of KIF21A self-regulation at the molecular detail, we first characterized the region of the KIF21A stalk, which was shown to mediate autoinhibition by interacting with the motor domain^12^,^13^. Sequence analysis of this region revealed two predicted coiled-coil segments (Supplementary Fig. S1a), one of them (aa938-1017) harbors all the CFEOM1-associated amino-acid residues of the stalk (Fig. 1). Based on this prediction, two KIF21A polypeptide chain fragments were prepared: rCC (aa938-1076), spanning the region containing both putative regulatory coiled coils and rCC1 (aa938-1017, regulatory coiled coil 1), corresponding to the first predicted coiled coil (Fig. 1).

**Figure 2:**
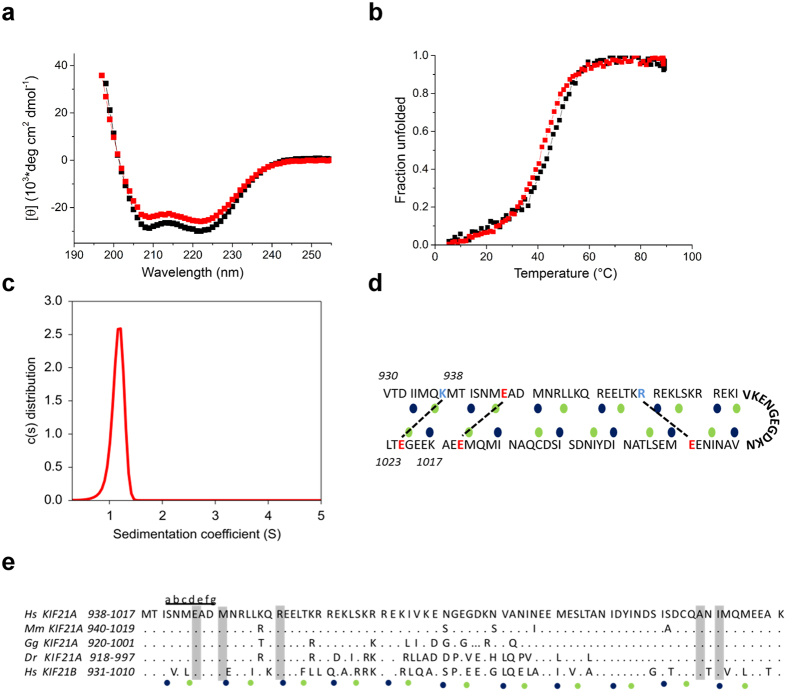
Biophysical characterization of the KIF21A stalk region mediating autoinhibition. (**a**) Far-UV CD spectra of rCC1 (red) and rCC1-L (black). CD measurements were performed in PBS at 5 °C using a protein concentration of 0.125 mg/ml. (**b**) Normalized thermal unfolding profiles of rCC1 (red) and rCC1-L (black) recorded by CD at 222 nm. Protein concentration and buffer conditions were as stated as in the legend of Fig. 2a. Global fitting yielded a T_m_ value of 42.4 °C for rCC1 and 45 °C for rCC1-L. (**c**) Oligomerization state of rCC1 determined by sedimentation velocity AUC at 20 °C and at a protein concentration of 500 μM. The theoretical and experimental M_W_ values are 9.5 and 12 kDa, respectively. (**d**) Model of the KIF21A intramolecular antiparallel coiled coil. Heptad repeats are shown as blocks of seven residues with heptad core *a* and *d* positions indicated by blue and green spheres, respectively. Charged amino acid residues are highlighted in color according to their physicochemical properties. Potential electrostatic interactions are indicated by dashed lines. The predicted loop is shown in bold. (**e**) Sequence alignment of selected KIF21A orthologues and hsKIF21B based on the model shown in panel d. Conserved amino acids are shown as dots, whereas not conserved amino acids are shown as one letter code. CFEOM1-associated amino acids are boxed (grey). Heptad repeats are shown as blocks of seven residues denoted *a* to *g*. Hs, Homo sapiens; Mm, Mus musculus; Gg, Gallus gallus; Dr, Danio rerio.

To assess the secondary structure content and the stability of rCC and rCC1, circular dichroism (CD) spectroscopy was performed. The far-ultraviolet CD spectra recorded for both polypeptide chain fragments revealed a significant amount of α-helical structure with distinct minima near 208 nm and 222 nm (Fig. 2a and Supplementary Fig. S1b). The stability of rCC and rCC1 was assessed by thermal unfolding profiles monitored by CD at 222 nm, which yielded nearly identical T_m_ values of ~43 °C (Supplementary Fig. S1c). Based on these very similar results, the biophysical characterization was continued with the shorter peptide, rCC1, only. To determine the oligomeric state of rCC1 in solution, sedimentation velocity analytical ultracentrifugation (AUC) (Fig. 2c) and size exclusion chromatography coupled to multi-angle light scattering (SEC-MALS, Supplementary Fig. S1d) experiments were performed, both demonstrating that rCC1 is monomeric even at concentrations as high as 500 μM. Together, these results are consistent with rCC1 forming an intramolecular antiparallel coiled coil.

Based on our biophysical findings we generated a structural model of the intramolecular antiparallel coiled coil (Fig. 2d). The fold is stabilized by the packing of residues at heptad repeat *a* and *d* positions against *d*’ and *a*’ residues, respectively, of the backfolding strand. The stability of the coiled coil might be further enhanced by two salt bridges (R960 and E988, K937 and E1021). The putative loop region contains two glycine residues (Gly976 and Gly 978), which are known to disrupt helical geometries^18^. The model is supported by a sequence alignment between KIF21A homologues and hsKIF21B, which illustrates that highly conserved residues, including the disease-related amino acids, are found predominantly at the termini of the antiparallel coiled coil (Fig. 2e).

Based on our model, we designed a third stalk fragment, rCC1-L (regulatory coiled-coil region 1- long; aa930-1023), which is slightly longer than rCC1 and therefore contains the second predicted salt bridge (K937 and E1021) not present in rCC1 (Figs 1 and 2d). Like rCC1, rCC1-L is a helical monomer in solution (Fig. 2a and Supplementary Fig. S1e), but revealed a ~2 °C higher T_m_ (Fig. 2b), suggesting that the potentially stabilizing salt bridge is formed in the structure. Taken together, these findings are consistent with our hypothesis that the KIF21A stalk region containing the CFEOM1-associated amino acids forms an intramolecular antiparallel coiled-coil domain.

### Crystal structure and small angle X-ray scattering of the KIF21A regulatory coiled coil

**Figure 3:**
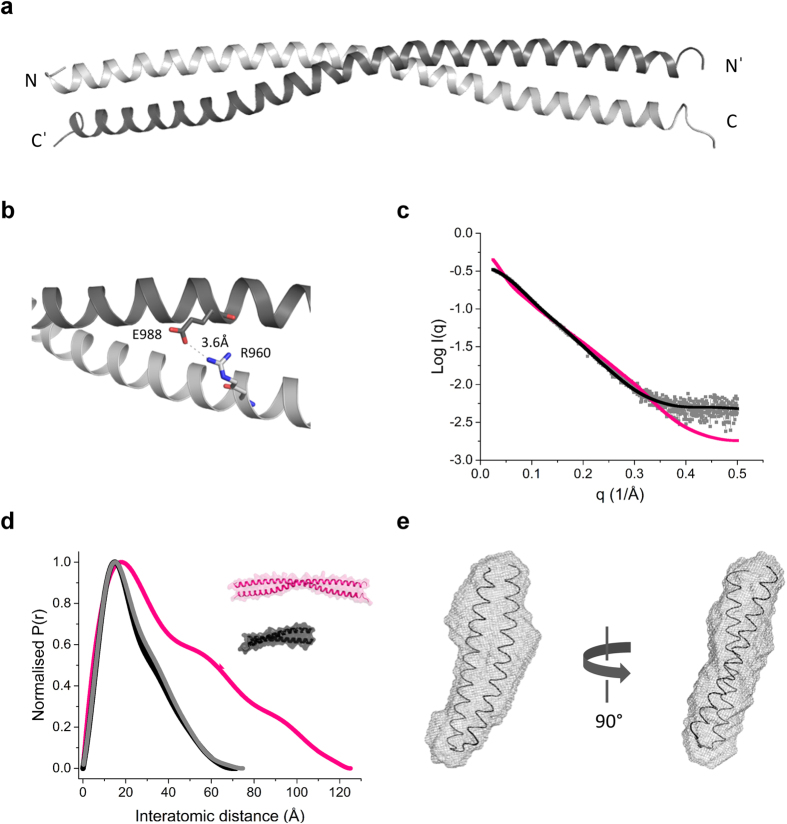
The regulatory domain of KIF21A folds into an antiparallel coiled coil as revealed by X-ray crystallography and SAXS. (**a**) Ribbon representation of the crystal structure of rCC1 showing a chain-swapped, antiparallel coiled-coil dimer. The two helices of the dimer are colored differently. Amino (N) and carboxy (C) termini are indicated. (**b**) Ribbon representation of the electrostatic interaction between R960 and E988 visible in the crystal structure of rCC1. The two helices of the dimer are colored as in panel a and the salt bridge distance is indicated. (**c**) Normalized distance distribution function for rCC1 (grey) and calculated distance distribution functions of the dimeric rCC1 crystal structure (rCC1-dimer, magenta) and rCC1-monomer (black). (**d**) Calculated scattering curve of rCC1 overlaid on the CRYSOL-derived scattering curves of rCC1-monomer and rCC1-dimer. (**e**) Two representative 90° rotated views of the rCC1-monomer model superimposed by Subcomb on the averaged and filtered DAMMIF model.

To obtain high-resolution structural information on the identified intramolecular antiparallel coiled-coil domain, all three coiled-coil fragments were subjected to extensive crystallization screening. However, only rCC1 yielded crystals suitable for structure determination. The crystal structure of rCC1 determined at 2.5 Å resolution is shown in Fig. 3a and the crystallographic parameters are listed in Table S1. Notably, rCC1 folds into a chain-swapped dimeric antiparallel coiled coil. Although being an intermolecular dimer, the two halves of this antiparallel coiled coil correspond to the heptad-repeat segments in our predicted model (Fig. 2d), including the predicted salt bridge formed by R960 and E988 (Fig. 3b). A plausible explanation for the observation of a chain-swapped dimer is provided by a detailed study reporting the biophysical characterization of an intramolecular, antiparallel coiled coil, similar to rCC1. Oakley *et al*. showed that the two individual moieties of the intramolecular antiparallel coiled coil of the seryl tRNA synthetase do not bind to each other or form homo- or heterotypic coiled coils in the absence of the connecting loop^19^. However, formation of the intramolecular antiparallel structure could be re-established by increasing the local concentration of the coiled-coil moieties through covalent connection of the two coiled-coil helices by an artificial disulfide bond. At maximal concentrations that can be reached in solution (typically a few mM) the antiparallel intramolecular coiled-coil conformation is favored. In the crystal, however, local concentrations of coiled-coil moieties are not a limiting factor anymore and an interaction of individual low-affinity coiled-coil moieties of different protein molecules will occur, explaining why a chain-swapped dimer is seen in our crystal structure.

To confirm this hypothesis, we used rCC1 to perform small angle X-ray scattering (SAXS) experiments, which provide information about the shape and size of a measured molecule in solution. The determined experimental mass of 8.8 kDa and the radius of gyration 19.5 (±1.6) Å obtained for rCC1 are consistent with the calculated theoretical values of 9.5 kDa and 19.2 Å, respectively, suggesting that the measured data are reliable. The shape of the scattering curve and the corresponding pair-distance distribution function, P(r), confirm that rCC1 is an extended molecule in solution with a maximal length, D_max_, of 75 Å (Fig. 3c,d). The pronounced peak seen in the P(r) centered at ~20 Å is in good agreement with the thickness of a two-stranded coiled coil. In a next step, we calculated the scattering curves and the P(r) functions from the obtained crystal structure, termed rCC1-dimer, and from the antiparallel rCC1 model, termed rCC1-monomer, in which the crystal structure was cut in half and the missing loop was modeled, and overlaid them with the experimental data of rCC1 (Fig. 3c,d). The dimeric antiparallel coiled-coil structure, rCC1-dimer, has a D_max_ at 125.1 Å, whereas rCC1-monomer reveals a D_max_ of 71.5 Å, which corresponds well to the experimental D_max_ of rCC1 (75 Å). In addition, the shapes of the P(r) functions of rCC1 and rCC1-monomer as well as the scattering curves are nearly identical, reinforcing our biophysical data showing that rCC1 is an intramolecular coiled coil in solution. Furthermore, *ab initio* shape reconstruction shows an elongated molecule (Fig. 3e) and overlay of the *ab initio* reconstructed envelope with rCC1-monomer provides further experimental support that the protein is a monomer in solution (Fig. 3e).

### The regulatory antiparallel coiled coil of KIF21A is an autonomous folding unit that is present in the full-length KIF21A stalk domain

**Figure 4:**
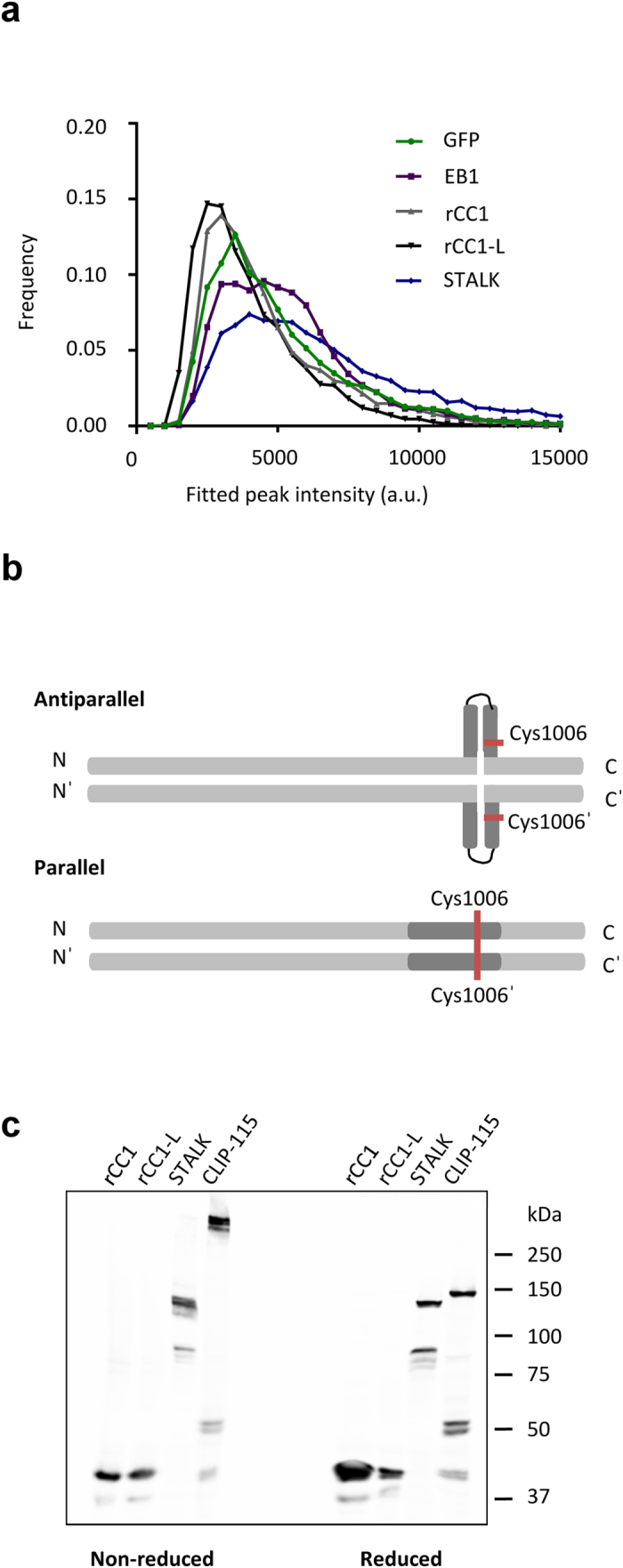
The intramolecular antiparallel coiled coil is an autonomous folding unit within the dimeric KIF21A stalk. (**a**) Distribution of initial (non-bleached) fluorescent intensities of single molecules. TIRF single molecule imaging was performed using HEK293T-cell extracts expressing indicated N-terminally biotinylated (Bio) GFP-tagged constructs. GFP and EB1-GFP serve as monomeric and dimeric controls, respectively^20^. (**b**) Schematic representation of two possible coiled-coil conformations of the KIF21A regulatory domain (dark grey) in the context of the KIF21A STALK (light grey). In the parallel conformation, Cys1006 (red) would be crosslinked to Cys1006′ of the opposite helix. In an intramolecular antiparallel conformation, Cys1006 would not be crosslinked. Amino (N) and carboxy (C) termini are indicated. (**c**) Cysteine crosslinking of GFP-labelled CLIP-115 and KIF21A stalk-domain fragments analyzed under reducing and non-reducing conditions by SDS-PAGE.

The regulatory coiled coil rCC1-L is flanked by regions that are not predicted to fold into coiled-coil structures (see also Fig. S1a). Therefore, rCC1-L corresponds to an autonomously folding antiparallel coiled-coil domain that is expected to adopt this fold also in the context of the full-length protein. KIF21A is known to form homodimers^12^, most likely through coiled-coil regions located N-terminal to the antiparallel coiled coil (Fig. 1). To confirm this assumption, the oligomeric state of the whole KIF21A stalk domain (termed STALK, Fig. 1) was investigated by single-molecule GFP-counting experiments. GFP and EB1-GFP were used as monomeric and dimeric controls, respectively^20^. The results show that STALK is dimeric, implying that the stalk domain is indeed responsible for KIF21A homodimer formation (Fig. 4a). They also confirmed our findings that rCC1 and rCC1-L are monomers in solution.

To demonstrate that the regulatory antiparallel coiled coil is formed in the context of the dimeric KIF21A stalk, cysteine crosslinking experiments were performed. Cys1006 is located at a hydrophobic heptad-repeat *d* core position and is expected to form a disulfide bond with Cys1006′ of the neighboring polypeptide chain in an in-register two-stranded parallel but not in an intramolecular antiparallel conformation (Fig. 4b). To exclude the first possibility, lysates of COS-7 cells expressing rCC1 and rCC1–L (monomeric negative controls), CLIP-115 (dimeric positive control^21^), and STALK fused to the C-terminus of monomeric GFP were analyzed by SDS-PAGE and Western blotting using anti-GFP antibodies. Under reducing conditions, all fusion proteins migrated at positions on the gel that correspond to their monomeric mass (Fig. 4c). Under non-reducing conditions, only the positive control, CLIP-115, formed a dimer of ~300 kDa while rCC1, rCC1-L and STALK remained monomeric (Fig. 4c). These findings strongly suggest that the regulatory antiparallel coiled coil is present in the context of the full-length KIF21A motor. Although we cannot rule out the possibility that STALK forms an antiparallel dimer, this conformation is very unlikely since the dimeric full-length KIF21A motor moves processively along MTs, suggesting a parallel head-to-head conformation^12^,^13^,^14^.

### The regulatory antiparallel coiled coil binds directly to the KIF21 motor domains and is sufficient for autoinhibition

**Figure 5:**
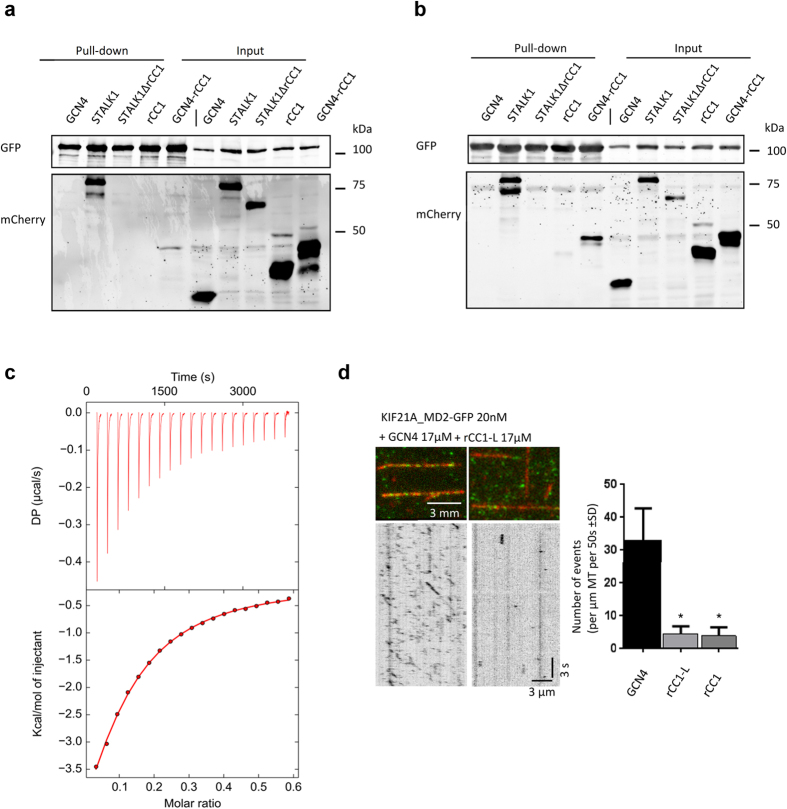
The regulatory antiparallel coiled coil binds to the KIF21A and KIF21B motor domains and is sufficient for KIF21A autoinhibition. (**a,b**) Streptavidin-based pull-down assay with lysates of HEK293T cells expressing the indicated proteins. Bio-GFP-tagged KIF21A_MD2 (**a**) and Bio-GFP-tagged KIF21B_MD1 (**b**) were used as baits. Coiled-coil polypeptide chain fragments are mCherry-tagged. (**c**) Binding affinity determined by ITC. rCC1 at a protein concentration of 900 μM was titrated into 48 μM solution of KIF21B_MD in 20 mM TrisHCl pH 7.5, 150 mM NaCl and 2 mM DTT and heat was measured at 10 °C. The ITC thermograph and fitted binding isotherm are shown in the upper and lower panel, respectively. (**d**) *In vitro* kinesin motility assay. Purified KIF21A MD2-GFP was added to taxol-stabilized MTs in flow chambers and analyzed for MT association in the presence of GCN4, rCC1 or rCC1-L. Both short MT binding and motile events were counted. Two independent measurements were analysed for rCC1, while 3 measurements were included for GCN4 and rCC1-L. (*P < 0.0001, Mann-Whitney U test).

The observation that all CFEOM1 mutations in the KIF21A stalk cluster in the N- and C-terminus of the regulatory coiled coil (Fig. 2) strongly supports an intramolecular antiparallel conformation of the fold and also suggests that this domain is the minimal region required for the interaction with its motor domain. To test this hypothesis, mCherry-labeled rCC1, a longer KIF21A stalk fragment, mCherry-STALK1, and mCherry-STALK1ΔrCC1 (Fig. 1), lacking the regulatory antiparallel coiled coil were assessed for their binding to the dimeric processive KIF21A motor domain, KIF21A_MD2, in pull down experiments^13^. As expected, STALK1 was able to bind to KIF21A_MD2, whereas deletion of the regulatory antiparallel coiled coil domain in STALK1 (STALK1ΔrCC1) completely abolished binding (Fig. 5a). However, an interaction of rCC1 with the dimeric KIF21A_MD2 could not be detected by pull down experiments. Since KIF21A was shown to be a dimer, we hypothesized that the binding to the motor domain might be stronger if rCC1 were dimeric. To test this idea, we engineered a dimeric rCC1-version, in which the parallel two-stranded GCN4 coiled coil^22^ was fused with a short linker to the N-terminus of rCC1 (termed GCN4_ rCC1). Indeed, we were now able to detect binding of mCherry-GCN4_rCC1 but not mCherry-GCN4 to KIF21A-MD2 (Fig. 5a). We furthermore showed that the C1006 in the recombinant GCN4_rCC1 does not crosslink under non-reducing conditions (Supplementary Fig. S2a), suggesting that the presence of the GCN4 fusion partner does not force the regulatory antiparallel coiled-coil domain into a parallel coiled coil. Together these experiments indicate the KIF21A antiparallel coiled coil itself is sufficient for binding to its motor domain.

To quantitate the interaction of the monomeric rCC1-variant with the KIF21A motor domain, we set out to determine the binding affinity of the two proteins *in vitro*. However, purification of KIF21A_MD yielded only low amounts of protein insufficient for binding studies. Therefore, we tested the highly similar KIF21B_MD, which shares 72% sequence identity with KIF21A_MD, including the three amino acids that are mutated in CFEOM1 (Supplementary Fig. S2b). Overall, recombinant KIF21B_MD behaved much better than KIF21A_MD in terms of expression levels and solubility. As expected, sedimentation velocity experiments at different concentrations revealed that the KIF21B_MD is monomeric in solution and able to bind MTs, suggesting that the protein is functional (Supplementary Fig. S2c–f). In a next step, we compared the binding properties of GFP-tagged KIF21B_MD1 to the mCherry-tagged KIF21A stalk fragments STALK1, STALK1Δ rCC1 and to rCC1 (Figs 1 and 5b). Notably, identical results were obtained for KIF21B and KIF21A motor domain constructs, demonstrating that KIF21B_MD is a suitable candidate for *in vitro* binding affinity studies. Accordingly, isothermal titration calorimetry (ITC) experiments using rCC1 revealed that it indeed binds with a low affinity to KIF21B_MD (Fig. 5c). Similar results were obtained for rCC (Supplementary Fig. S2g). Low affinity binding between isolated autoinhibitory domains of kinesins was also described for the KIF5 head-tail interaction^23^.

Previously, it has been shown that the stalk of KIF21A is able to inhibit its motor activity^12^. To validate that the regulatory antiparallel coiled coil alone is sufficient for the inhibition of the motor, we performed *in vitro* motility assays with KIF21A_MD2. As a control, we used the parallel coiled coil of GCN4, which does not bind to MD2 (Fig. 5a,b). In the presence of GCN4, MD2 was processive and displayed numerous MT-association events (Fig. 5d). In contrast, the addition of an excess of recombinant rCC1 or rCC1-L resulted in a significant decrease of MT-association events, demonstrating that the interaction of the rCC1 and the motor domain is sufficient for inhibition, even when the two domains are not present in the same polypeptide chain. Collectively, these findings demonstrate that the intramolecular antiparallel coiled coil of KIF21A is the minimal autoinhibitory domain. Because the full-length KIF21A protein exhibits similar autoinhibition, these functional studies also provide strong evidence for the existence of the regulatory antiparallel coiled-coil domain in the context of the full-length KIF21A protein. Furthermore, we show that the KIF21A antiparallel coiled coil interacts with the KIF21B motor domain, suggesting a conservation of the autoinhibitory mechanism in KIF21A and KIF21B.

### CFEOM1 mutations relieve autoinbibition through different mechanisms

**Figure 6:**
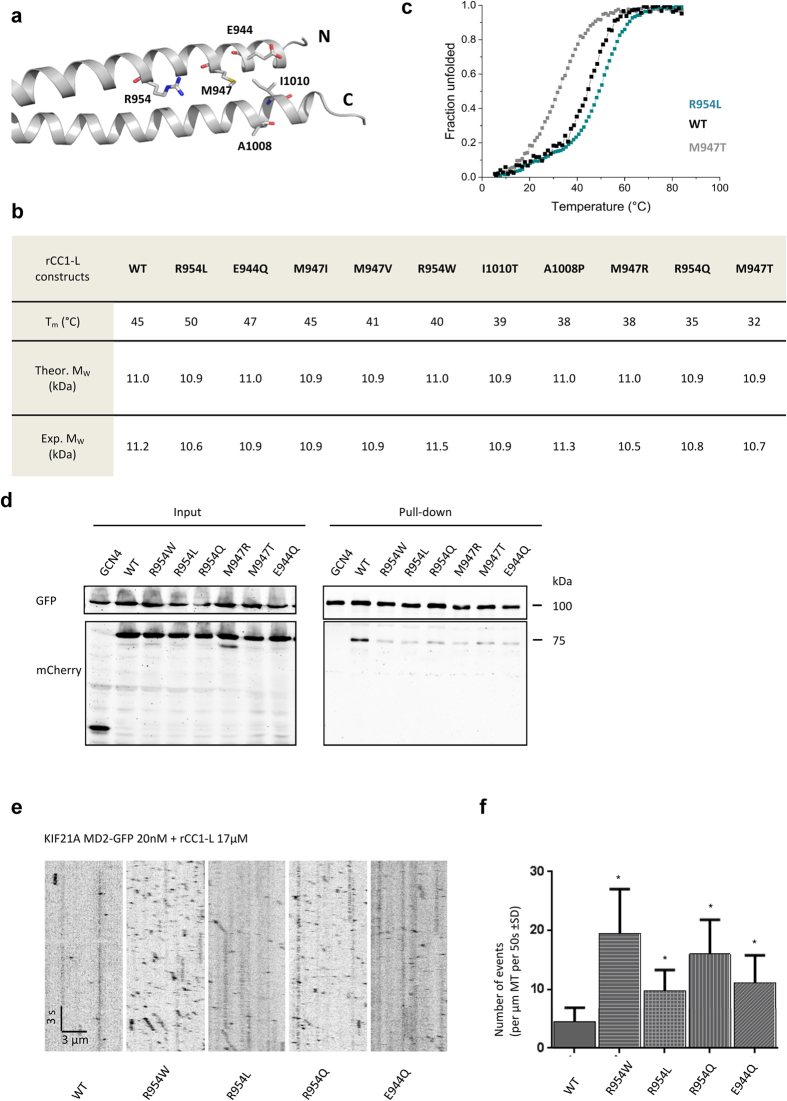
CFEOM1 mutations release KIF21A autoinhibition by destabilizing the regulatory antiparallel coiled coil or by affecting residues of the binding interface. (**a**) Positions of the CFEOM1-associated amino-acid residues shown in one half of the KIF21A rCC1 crystal structure. Amino (N) and carboxy (C) termini are indicated. (**b**) Summary of the biophysical characterization of wild-type rCC1-L and its CFEOM1 mutants by thermal unfolding monitored by CD at 222 nm using a protein concentration of 0.125 mg/ml and SEC-MALS performed at a protein concentration of 2 mg/ml. (**c**) Normalized thermal unfolding profiles of wild-type rCC1-L and its most destabilizing (M947T) and most stabilizing mutants (R954L). (**d**) Streptavidin-based pull-down assay with lysates of HEK293T cells expressing Bio-GFP-tagged KIF21A_MD2 used as bait for the pulldown of mCherry-labelled STALK1_WT and mutant polypeptide chain fragments. (**e**) *In vitro* kinesin motility assay. The assay was performed as described in Fig. 5d, but in the presence of wild-type or mutant rCC1-L domains. Values significantly different from the wild-type control are indicated by an asterisk (P < 0.0001, Mann-Whitney U test).

To date, ten CFEOM1 mutations and a one-residue deletion have been identified in the stalk of KIF21A, affecting six residues (E944, M947, R954, D1001, A1008, I1010) (Figs 1 and 6a^15^,^24^,^25^). To understand the molecular mechanism of how CFEOM1-associated mutations affect KIF21A autoinhibition, we biophysically and biochemically characterized rCC1-L variants containing individual CFEOM1 point mutations. Notably, none of the mutations resulted in a change of the oligomerization state compared to wild-type rCC1-L as demonstrated by SEC-MALS (Fig. 6b). However, thermal unfolding monitored by CD at 222 nm revealed substantial differences in the stabilities of the mutants (Fig. 6b,c). Apart from M947I, which has an identical T_m_ as wild-type rCC1-L, the majority of the mutations had a destabilizing effect when compared to the wild-type peptide. Because some of the mutants showed a fraction of 50% unfolded molecules in solution at physiological temperature (Fig. 6b,c), their impact on autoinhibition can be rationalized in terms of reduced binding to the motor domain as a result of destabilization of the regulatory antiparallel coiled-coil structure. This is for example the case for the disease-causing point mutation M947R, where the introduction of a charged side chain at a hydrophobic core heptad-repeat *a* position results in decreased stability of the domain. Only two rCC1-L mutants, R954L and E944Q, showed an increase in thermal stability relative to the wild type. In R954L, the charged arginine located at a hydrophobic core heptad *a* position is mutated to leucine, the most common and most stabilizing hydrophobic residue found in coiled coils, whereas in E944Q, the glutamate forming a repulsive ionic interaction with E1014 of the opposite chain (Fig. 2d) is mutated to a polar glutamine. Since these mutations do not destabilize the regulatory antiparallel coiled coil or change its oligomerization state, their effect on KIF21A autoinhibition can be best explained by the involvement of the corresponding side chains in the binding to the motor domain.

To confirm that differences in regulatory antiparallel coiled-coil stability influence the binding to the motor domain, pull down assays were performed with KIF21A_MD2 and variants of STALK1 representing two stabilizing and four destabilizing mutations. HEK293T cell lysates expressing the motor domain in combination with a stalk fragment variant revealed similar reduced binding of all mutants to KIF21A_MD2 when compared to the wild type (Fig. 6d). These results confirm and complement earlier findings^12^,^13^ where two CFEOM1-associated stalk mutations (M947I and R954W) were shown to result in a reduced binding to KIF21A MD compared to the wild-type protein. In a next step, we compared the MT binding of KIF21A_MD2 in the presence of rCC1-L wild type and mutant polypeptides in a more sensitive *in vitro* motility assay. The number of MT binding events of KIF21A_MD2 in the presence of rCC1-L mutants was substantially higher as compared to the rCC1-L wild type fragment (Fig. 6e,f).

Taken together, these data show that neither of the CFEOM1 point mutations alters the oligomeric state of the regulatory antiparallel coiled coil. The mutations either decrease the stability of the regulatory coiled coil or affect side chain-side chain interactions in the binding interface to the motor domain. In addition, the tested rCC1-L mutants displayed a reduced ability to inhibit the motor as compared to the wild type.

### Molecular mechanism of KIF21A autoinhibition based on molecular docking and mutagenesis

**Figure 7:**
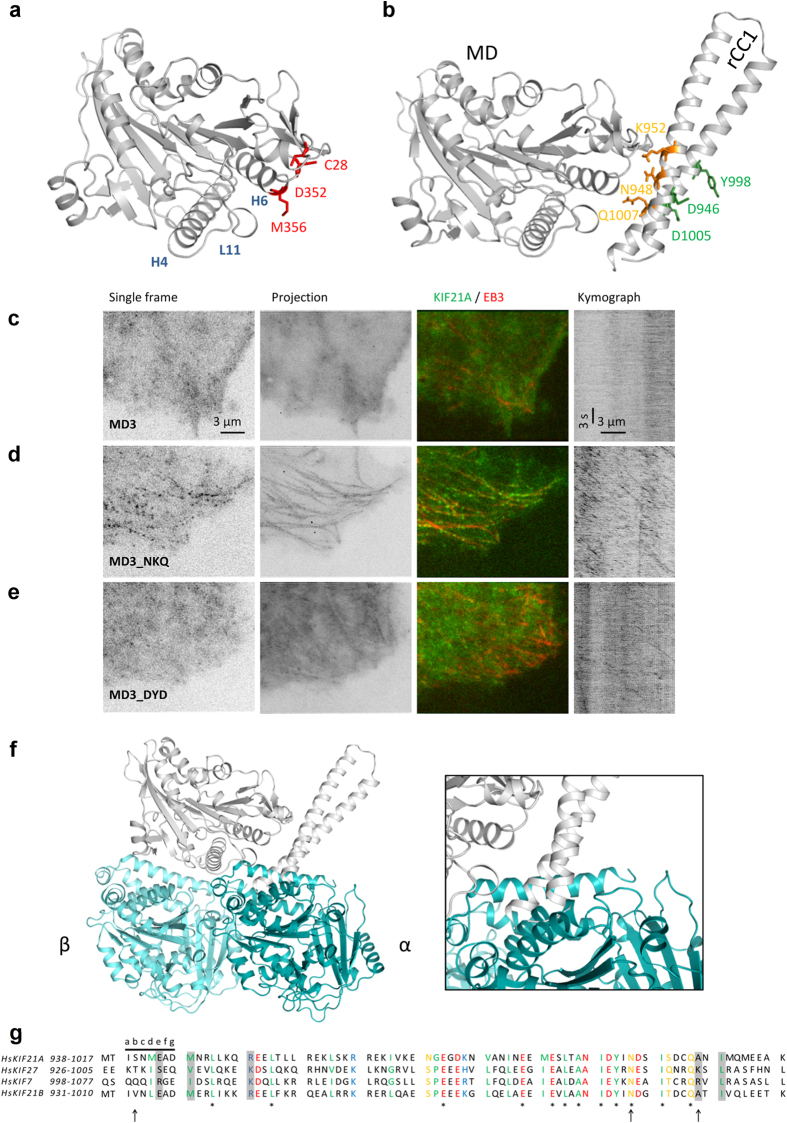
Molecular docking of the KIF21A motor domain and the regulatory antiparallel coiled coil. (**a**) KIF21A homology model based on the KIF4 motor-domain structure (PDB code 3ZFD). The positions of CFEOM1 mutations in the motor domain are indicated. The α-tubulin subunit interacting elements are labeled in blue on the basis of the KIF5-tubulin complex crystal structure (PDB code 4HNA^30^). (**b**) Docking model (light grey) with residues targeted for mutagenesis highlighted. The NKQ residues involved in the interface and the DYD residues pointing away from the interface (negative control) are shown in orange and green, respectively. (**c–e**) TIRFM-based live cell imaging of COS-7 cells transiently transfected with KIF21A_MD3 or the two mutant versions of this construct, NKQ or DYD, fused to GFP. Panels from left to right: a single frame of the GFP channel; maximum intensity projection of the GFP channel over 500 frames (50 s), and a kymograph along a single MT, illustrating the motile behavior of KIF21A_MD3-NKQ and very limited motility of the wild-type KIF21A_MD3 and KIF21A_MD3-DYD. (**f**) Superposition of the KIF5-tubulin complex (dark green α-tubulin, light green β-tubulin) crystal structure (PDB code 4HNA^30^) and the KIF21A docking model (grey) indicates the mechanistic details of KIF21A autoinhibition. (**g**) Sequence alignment of the regulatory antiparallel coiled coil in hsKIF21A with the corresponding sequences in hsKIF21B, hsKIF7 and hsKIF27. Conserved amino-acid residues are marked by an asterisk. Similar hydrophobic (green), polar (orange), positively charged (blue) and negatively charged (red) amino acids are depicted. Amino acids known to be mutated in patients with Bardet-Biedl syndrome are indicated by arrows. CFEOM1-associated amino acids are highlighted (grey). Heptad repeats are shown as blocks of seven residues denoted *a* to *g*.

Three CFEOM1 mutations, C28W, M356T and the recently identified D352E, are found in the motor domain of KIF21A (Fig. 1)^26^. Figure 7a shows the location of these three residues in a homology model of the KIF21A motor domain (KIF21A_MD1). Notably, all of these mutations cluster at the same site of the motor domain, very close to the α-tubulin interacting helix H6 and loop L11^27^,^28^. Previously, we and others have shown that the introduction of C28W and M356T into the motor domain abolished binding to the coiled-coil stalk but did not affect its structural integrity as judged by MT binding and motility of the motor^12^,^13^. This observation strongly suggests that this site is part of the binding interface with the regulatory antiparallel coiled coil. Based on this hypothesis, the crystal structure of rCC1-monomer and the KIF21A_MD1 homology model were used to generate a docking model of the complex^29^ (Table S2). E944 and R954 in the antiparallel coiled coil as well as M356 and C28 in the motor domain were chosen as interface residues for the protein-protein docking. The ensemble of the four best structures from the top cluster is shown in the Supplementary Fig. S3a. The model predicts that the rCC1 domain makes predominantly electrostatic interactions with the motor domain residues in helix α6, α0, in the loop connecting β1b and β1c, L2 as well as with L11, resulting in a 1308.6 Å^2^ protein-protein binding interface (Fig. 7b and Supplementary Fig. S3b)^27^,^28^. According to the docking model, M356 interacts with the CFEOM1-associated residue M947 in the stalk, which may explain why mutation M947 to isoleucine causes autoinhibition release without resulting in a stability change of this rCC1-L mutant when compared to the wild-type domain (Fig. 6b). Although both residues are hydrophobic, isoleucine is a bulkier β-branched residue when compared to methionine, suggesting that side chain geometry could be a critical factor for the interaction with M356 of the motor domain.

To validate the docking model, we mutated three residues, N948A, K952A and Q1007A (subsequently named NKQ), which are predicted to participate in the interaction between the motor domain and the regulatory antiparallel coiled coil. In addition, these residues were chosen to occupy heptad-repeat positions outside the hydrophobic core to avoid destabilization of the antiparallel coiled coil (Figs 2d and 7b). A second triple mutant (subsequently named DYD) was engineered to serve as a negative control, in which residues outside the regulatory antiparallel coiled coil-motor domain interface and outside the hydrophobic core of the coiled coil were selected (Figs 2d and 7b). As expected, both triple mutations did not affect the oligomerization state of rCC1-L as judged by SEC-MALS (Supplementary Fig. S3c). Even more important, both triple mutants did not change the secondary structure nor destabilize rCC1-L as seen by CD spectra and CD unfolding experiments, respectively (Supplementary Fig. S3d,e). Subsequently, both triple mutation sets were introduced in the autoinhibited GFP-tagged construct KIF21A_MD3 and tested for their ability to release autoinhibition upon transfection into cells (Fig. 7c)^13^. While NKQ mutations resulted in a motile KIF21A motor as visualized by GFP fluorescence signal along MTs (Fig. 7d), DYD mutations had a much milder effect on the KIF21A_MD3 autoinhibition (Fig. 7e). Together, these results support the validity of our docking model.

To reveal additional details on how autoinhibition might prevent KIF21A motors from MT- binding, we superimposed our docking model onto the tubulin-KIF5 structure (Fig. 7f)^30^. Strikingly, the comparison revealed that rCC1 appears to prevent binding of the motor domain H6 and L11 segments to α-tubulin (Fig. 7f). Of particular interest is the interaction of rCC1 with L11. In the free kinesin state, L11 is disordered; however, upon MT binding the loop becomes structured and thereby helps accelerating the ATP hydrolysis and processivity of the motor^30^. In this context, it is also noteworthy that the CFEOM1-associated residue M356 in the motor domain is located in H6 of the KIF21A motor domain. Very likely M356 is a critical residue for MT-binding since it cor motor domain - coiled coil responds to S309 of KIF5, which forms a hydrogen bond with E420 of the α-tubulin subunit^30^. Our docking model reveals that the M356 is also involved in binding to the regulatory antiparallel coiled coil domain by establishing a hydrophobic interaction with M947. The M356T mutation might, therefore, decrease the motor affinity for the regulatory antiparallel coiled coil domain and increase the affinity to tubulin, thus shifting the equilibrium towards an active conformation of KIF21A.

In summary, our proposed docking model provides a rational explanation how the regulatory antiparallel coiled-coil element autoinhibits binding of KIF21A to MTs.

## Discussion

Kinesin motor proteins must be tightly regulated in the absence of cargo to prevent the emergence of pathologies such as neurological disorders. One important regulatory mechanism is kinesin autoinhibition, for which several different types have been described. Typically, kinesin autoinhibition is achieved by the interaction of a motor domain with one copy of at least one regulatory domain. To our knowledge, this is the first study, in which an intramolecular antiparallel coiled coil is reported as a regulatory domain of a kinesin motor.

We found that the KIF21A regulatory antiparallel coiled coil also binds the related KIF21B motor domain. Because it was shown that KIF21A and KIF21B do not form heterodimeric motors^14^, this finding strongly suggests that a similar autoinhibitory mechanism is present in both KIF21A and KIF21B. This hypothesis is supported by structure-based sequence alignments of hsKIF21A and hsKIF21B motor domains and regulatory regions (Fig. 2e and Supplementary Fig. S2a), which demonstrate sequence identities of 45% and 75%, respectively. Furthermore, all CFEOM1-associated residues in the stalk and the motor domain are conserved among the two proteins. Together, these observations are consistent with the presence of a regulatory antiparallel coiled coil in the stalk of KIF21B.

Apart from the two highly homologous KIF21A and KIF21B proteins, the kinesin-4 family includes the chromokinesin KIF4 and the ciliary associated motors KIF7 and KIF27^13^,^31^,^32^. To assess if other kinesin-4 family members share a similar autoinhibition mechanism with KIF21A, a BLAST search was performed using the sequence of the rCC1 domain as input sequence. In this search, two other members of the kinesin-4 family, KIF7 and KIF27, were identified as potential candidates sharing 37.5% sequence similarity to the KIF21A rCC1 domain (Fig. 7g). Notably, the predicted KIF7 and KIF27 sequences are found in their C-terminal stalk and both sequences are also predicted to fold into coiled coils. Although the CFEOM1- associated amino acid residues are not conserved in KIF7 and KIF27, four and three out of five disease related residues in KIF7 and KIF27, respectively, retained amino acid similarity in terms of their physicochemical properties (Fig. 7g). Consistent with the preservation of an antiparallel coiled-coil structure, the predicted loop regions of KIF7 and KIF27 contain helix-breaking residues.

KIF7 is related to many neurological diseases, such as hydrolethalus, acrocallosal and Bardet- Biedl syndromes^33^,^34^. Interestingly, a disease-causing point mutation (N1060S), a nonsense mutation (Q1001X) and a heterozygous point mutation R1068W all lie in the region with highest sequence similarity to the KIF21A rCC1 domain. Remarkably, one of them is located at a CFEOM1-affected position and is also a R to W amino acid substitution, the same as found in the most common CFEOM1-associated mutation in KIF21A (Fig. 7g). To address whether diseases like Bardet-Biedl syndrome are associated with aberrant kinesin autoinhibition, it would be important to clarify if KIF7 and KIF27 share a similar autoinhibitory mechanism as KIF21A.

Different regulatory mechanisms of autoinhibition release are found in kinesin motors (reviewed by^7^). The kinetochore motor CENP-E, for example, is activated upon phosphorylation of the regulatory tail domain by MPS1- or CDK1 kinases^35^, whereas KIF5 autoinhibition is released by binding of its cargo proteins c-Jun N-terminal kinase–interacting protein 1 (JIP1) and fasciculation and elongation protein ζ1 (FEZ1)^36^.

An interesting, yet unresolved question is how KIF21A autoinhibition is naturally regulated. Several binding partners of KIF21A have been identified, including the neuronal MT-associated protein Map1b^12^, brefeldin A-inhibited guanine nucleotide-exchange protein (BIG) 1^37^, the cortical adaptor protein KANK1^13^,^38^ and the K(+)-dependent Na(+)/Ca(2+) exchanger (NCKX)^39^. Notably, all these proteins interact with C-terminal elements of KIF21A. For example, Map1b is interacting with the KIF21A WD40 and stalk region containing the rCC1 domain^12^. It should therefore be of high interest to investigate whether and how the proteins contribute to the release of KIF21A autoinhibition.

In this context, it should also be noted that the ubiquitin E3 ligase, TRIM3, was shown to regulate KIF21B motility *in vivo*^40^. Interestingly, TRIM3 binds to a region of the KIF21B stalk containing the potential rCC1 domain^40^. Recent findings show that KIF21B is genetically linked to Alzheimer’s disease and multiple sclerosis^41^. Knowledge on the regulation of KIF21B function therefore appears important for contributing towards understanding these human diseases.

Taken together, we identified a novel autoinhibitory mechanism for kinesin KIF21A, which might also be present in other kinesin-4 family members. Detailed knowledge on the regulation of motor activity could contribute towards understanding the mechanistic details underlying several severe human diseases, including Alzheimer’s and multiple sclerosis.

## Methods

### Cloning and protein production

cDNA fragments encoding KIF21A rCC (residues 938–1076), rCC1 (residues 938–1017), and KIF21B_MD (residues 1–369) were PCR amplified from a human cDNA library^42^ and cloned into the pET-based bacterial expression vector PSTCm1^43^. KIF21A rCC1–l (residues 930–1023) was PCR amplified from a synthetic gene fragment optimized for expression in E. coli and cloned into the pET15b-based expression vector pHisTrx^44^. Mutant variants of rCC1-L were generated by PCR amplification of the whole plasmid using primers containing the mutation^43^. All proteins were expressed in BL21 (DE3) (New England Biolabs) except KIF21B_MD was expressed in Rosetta 2 (DE3) (Novagen). Cells were grown at 37 °C in LB media supplemented with either 100 μg/ml ampicillin or a mixture of 50 μg/ml kanamycin and 30 μg/mL chloramphenicol to an OD_600_ of 0.4–0.6. After reduction of the temperature to 20 °C protein expression was induced with 0.5 mM isopropyl 1-thio-β- galactopyranoside (IPTG, Sigma-Aldrich) and incubation was continued overnight. The cells were harvested by centrifugation. The cell pellets were resuspended in lysis buffer (50 mM HEPES pH 8, 500 mM NaCl, 10 mM Imidazole, 10% glycerol, 2 mM β-mercaptoethanol and 1 cOmplete EDTA-free protease inhibitor cocktail tablet (Roche). The cells were lysed on ice by ultrasonication. Lysate clearing was performed for 30 min at 30,000 rpm, and the resultant supernatant was filtered (0.45 μm filter). The proteins were subsequently purified at 4 °C by IMAC (on a 5 ml HisTrap FF Crude column, GE Healthcare) according to manufacturer’s instructions.

The proteins were dialyzed against thrombin cleavage buffer (20 mM TrisHCl pH 7.4, 150 mM NaCl, 2.5 mM CaCl2 and 2 mM DTT) and the 6xHis tag or the 6xHis-tagged thioredoxin fusion protein were removed by overnight digestion at 4 °C using 2 units of human thrombin (Sigma-Aldrich) per milligram of recombinant protein. The 6xHis tag or the 6xHis-tagged thioredoxin fusion proteins were separated from the target proteins by re-application to the IMAC column. Target proteins were concentrated and further purified by gel filtration on a HiLoad Superdex 200 16/60 size-exclusion column (GE Healthcare) equilibrated in 20 mM TrisHCl, pH 7.5, supplemented with 150 mM NaCl and 2 mM DTT.

KIF21B-MD was purified as described above without removing the 6xHis tag. The homogeneity of the recombinant proteins was assessed by acrylamide PAGE and their identity was confirmed by ESI-TOF mass spectrometry.

### Biophysical characterization

SEC-MALS experiments were performed in 20 mM TrisHCl, pH 7.5, 150 mM NaCl, 2 mM DTT buffer using a S-200 13/10 analytical size-exclusion chromatography column (GE Healthcare) connected in-line to mini-DAWN TREOS light scattering and Optilab T-rEX refractive index detectors (Wyatt Technology). Proteins were injected at a concentration of 2 mg/ml.

CD spectra were recorded at 5 °C and at a protein concentration of 0.125 mg/ml in PBS supplemented with 0.5 mM TCEP using a Chirascan spectropolarimeter (Applied Photophysics) and a cuvette of 0.1 cm path length. Thermal unfolding profiles between 5 °C and 80–90 °C were recorded by increasing the temperature at a ramping rate of 1 °C/min monitoring the CD signal at 222 nm. Midpoints of thermal unfolding were calculated using the Glob3 program (Applied Photophysics).

AUC sedimentation velocity experiments were performed in Tris-HCl, pH 7.5, 150 mM NaCl, 2 mM DTT using a Beckman XLI analytical ultracentrifuge (Beckman Coulter). Samples were measured at different protein concentrations using 20 °C, 42000 rpm. Sedimentation profiles were recorded by UV absorbance (280 nm) and interference scanning optics. The partial specific volume of the samples as well as the density and viscosity of the buffer were calculated with SEDNTERP. Data were fitted with Sedfit using the continuous distribution model and figures were processed with GUSSI (biophysics.swmed.edu/MBR/software.html).

### Crystallization and structure elucidation

rCC1 (22 mg/ml) was crystallized by sitting drop vapour diffusion at 20 °C in citric acid, pH 4.00 and 10% (w/v) PEG 6000. For cryoprotection, the reservoir solution was supplemented with 30% glycerol. A complete 2.5 Å resolution data set was collected from a single crystal at 100 K at the X06DA beamline of the Swiss Light Source (Paul Scherrer Institute) using a wavelength of 2.066 Å. The data was reduced, scaled, merged and converted with XDS, XSCALE and XDSCONV^45^. The structure of rCC1 was solved using the ROSETTA^46^ *ab initio* modeling-based, automated molecular replacement pipeline AMPLE^47^. 3-amino acid and 9-amino acid peptide fragments for the *ab initio* modeling step were generated with the ROBETTA web server^48^. Molecular replacement was performed with PHASER^49^. Although the space group P622 was suggested by XTRIAGE^50^, clashes with supposed symmetry mates and high Rwork/Rfree (0.26/0.32) suggested twinning. The structure was solved in space group P3121 allowing for the direct refinement of the dimeric interface using the twin law -h,-k,l. Initial model building was done using BUCCANEER^51^. The structure was refined using Phenix.refine from the Phenix suite^52^ and the program COOT for manual real space refinement^53^. The model quality was evaluated with MOLPROBITY^54^. The structure was deposited in the PDBe under the following accession code: 5D3A.

### SAXS data collection and analysis

SAXS data were collected at the cSAXS (X12SA) beamline at the Swiss Light Source (Paul Scherrer Institute) and experiments were performed at 10 °C as described previously^55^. SAXS data analysis of the integrated and subtracted scattering data was done in parallel with the ATSAS software suite^56^ and with an in house software package. The merging of the buffer subtracted scattering data was performed with PRIMUS^57^. Distance distribution functions were derived from the experimental data with GNOM^58^. They were used as input for the DAMMIF/DAMMIN *ab initio* shape reconstruction in interactive mode^59^,^60^. 10 initial DAMMIF runs were averaged and filtered with DAMAVER^61^ and refined with 10 subsequent DAMMIN runs. Theoretical scattering curves for the crystal structure of the KIF21A coiled coil (rCC1-dimer) and the rCC1-monomer model were calculated with CRYSOL^62^ using default parameters. From those theoretical scattering curves the theoretical distance distributions were derived with GNOM.

### Isothermal titration calorimetry (ITC)

ITC data were collected using a MicroCal iTC200 (MicroCal, Northampton, Massachusetts, United States). Binding of rCC1 and rCC to KIF21B_MD was determined in 20 mM TrisHCl, pH 7.5, 150 mM NaCl at 10 °C. KIF21B_MD in the cell (45–60 μM) and rCC1 or rCC in the syringe (900–1000 μM) were dialyzed in the above mentioned buffer. Titrations were performed at 0.2 min spacings at a stirring speed of 1000 s^−1^. Data were integrated with NITPIC^63^ and isotherms were fitted using a 1:1 bimolecular interaction model with SEDPHAT^64^. Final images were done with GUSSI (biophysics.swmed.edu/MBR/software.html).

### MT pelleting assay

For the MT pelleting assay, 10 mg/ml tubulin stock was diluted to 1.1 mg/ml in BRB80 buffer (80 mM K-PIPES, pH 6.8, EGTA 1 mM, MgCl_2_ 1 mM) supplemented with 0.5 mM GTP and 1.25 mM DTT and kept on ice for 5 min. MT polymerization was initiated by incubating the tubulin mix for 10 min at 37 °C followed by a stepwise addition of 0.1 μM, 1 μM and 10 μM paclitaxel (dissolved in DMSO (Sigma Aldrich)) with 5 min incubation intervals between each step and final incubation step of 10 min at 37 °C. 3 μM polymerized MTs were incubated with 2.5 μM KIF21B_MD diluted in BRB80 for 20 min at 20 °C. MTs alone and KIF21B_MD alone at the same concentrations were included as controls. A taxol-glycerol cushion containing 55% 2x BRB80, 44% glycerol and 6% 2 mM paclitaxel was added to the centrifugation tubes prior to sample addition (Sigma-Aldrich). After centrifugation at 174,500 × g for 25 min, 10 μl of the supernatant was carefully removed and added to 2.5 μl 5x SDS loading dye. The remaining supernatant was discarded and the pellets were resuspended in 100 μl BRB80 buffer with 25 μl 5x SDS loading dye. Supernatant and pellet fractions (12.5 μl) were analyzed by 12.5% SDS-PAGE.

### Cell culture, DNA transfection and constructs

Cos-7 and HEK293T cells were cultured in DMEM/F10 (1/1 ratio, Lonza) supplemented with 10% fetal calf serum and penicillin and streptomycin. KIF21A expression constructs were generated as described previously^13^. Plasmids were transfected with polyethylenimine (PEI).

### GFP counting assay

Lysates of HEK293T cells expressing Bio-GFP, EB1-GFP and KIF21A proteins fused to a monomeric GFP were prepared in 20 mM Tris pH7.5, 100 mM NaCl, 1% Triton-X100, 1 x cOmplete protease inhibitor cocktail tablet (Roche) and added to the imaging flow chambers made from plasma cleaned coverslips. After washing with PBS, a significant fraction of GFP-fused molecules were immobilized on the coverslip. Each lysate was diluted to 400–800 individual molecules per field of view (30 μm × 30 μm). GFP molecules were imaged at room temperature by TIRF microscopy, using an inverted Nikon Eclipse Ti-E (Nikon) research microscope equipped with the perfect focus system (PFS) (Nikon), a Nikon CFI Apo TIRF 100 × 1.49 N.A. oil objective (Nikon), an ET-GFP filter set (Chroma Technology Europe) for imaging of GFP-tagged proteins, a 491-nm 100-mW Calypso (Cobolt, Solna) laser, a Photometrics Evolve 512 EMCCD (Roper Scientific) with an intermediate 2.5× lens (Nikon C mount adapter 2.5×), TIRF-E motorized TIRF illuminator modified by Roper Scientific France/PICT-IBiSA Institut Curie and controlled by the MetaMorph 7.7 software (Molecular Devices). A set of 20–30 images per condition with 100-ms exposure was acquired ‘blindly’ at different places of the coverslip to avoid pre-bleaching. Detection and analysis of single molecules were performed using the ImageJ plugin DoM_Utrecht as described previously^65^. Histograms of GFP intensities were built using GraphPad Prism version 6 for Windows.

### Cysteine crosslinking assay

COS-7 cells transfected with KIF21A constructs fused to the C-terminus of GFP were lysed in 1x PBS containing 1% Triton-X100, 1x cOmplete protease inhibitor cocktail tablet (Roche) and incubated for 1 h at room temperature. Samples were run by SDS-PAGE under reducing (+DTT) and non-reducing conditions (-DTT) and analyzed by Western blotting. Recombinant GCN4_rCC1, rCC1 and Bld-10 were diluted 1:100 in buffer with or without reducing agent, let incubate for 30 min and mixed with 5x loading dye with or without 2 mM β-ME. Samples were run on SDS PAGE and stained with Coomassie blue.

### Pull down assays

HEK293T cells were transfected with Bio-GFP-tagged constructs as bait, which contain a linker encoding the sequence for the substrate of biotin ligase BirA, MASGLNDIFEAQKIEWHEGGG, and mCherry-tagged prey proteins. The cells were lysed in 20 mM Tris pH7.5, 100 mM NaCl, 1% Triton-X100, 1 cOmplete protease inhibitor cocktail tablet (Roche). The lysates were incubated with M-280 Streptavidin Dynabeads (Invitrogen) for one hour and subsequently the beads were washed. After the addition of sample buffer containing +DTT samples were run by SDS-PAGE and subsequently analyzed by Western blotting. The BirA ligase expression construct used in this study was a gift from D. Meijer (Erasmus MC, Rotterdam, The Netherlands).

### *In vitro* motility assay

*In vitro* motility assays were performed using flow chambers, MTs and isolated KIF21A protein as described previously^13^. The MTs were attached to a coverslip via biotin-NeutrAvidin links and incubated with 0.8 mg/ml κ-casein. The reaction mix consisting of 20 nM KIF21A MD2-GFP, 17 μM GCN4 or 17 μM wild-type rCC1-L or 17 μM rCC1-L mutants and an oxygen scavenging system (200 μg/ml catalase, 400 μg/ml glucose-oxidase, 4 mM DTT), 10 mM glucose, 2 mM ATP, 2 mM MgCl2 in MRB80 buffer was added to the flow chambers. Samples were imaged at 30 °C by TIRF microscopy as described previously^13^. Data were analyzed using ImageJ and GraphPad Prism 6.

### Homology modelling and molecular docking

Homology models of the KIF21A motor domain (termed KIF21A_MD1) were generated with PHYRE2 (**P**rotein **H**omology/analog**Y R**ecognition **E**ngine V 2.0) using the related KIF4 motor domain crystal structure (PDB: c3zfcA) as a template.

Docking was performed using the protein interface prediction HADDOCK 2.2 webserver. E944 and R954 were chosen as active residues for the rCC1-monomer and M356 and C28 were selected as active residues for the KIF21A motor domain homology model. Passive residues were defined automatically around the active residues. 10 clusters, ranked according to their HADDOCK scores, were generated with the top two clusters having a score of -122.1 (cluster 1) and -118.5 (cluster 4), respectively (Table S2).

## Additional Information

**How to cite this article**: Bianchi, S. *et al*. Structural basis for misregulation of kinesin KIF21A autoinhibition by CFEOM1 disease mutations. *Sci. Rep*. **6**, 30668; doi: 10.1038/srep30668 (2016).

## Supplementary Material

Supplementary Information
