# Supplementary material for: Structural basis for misregulation of kinesin KIF21A autoinhibition by CFEOM1 disease mutations
Source: Sci Rep. 2016 Aug 3;6:30668. doi: 10.1038/srep30668 (PMC4971492; doi:10.1038/srep30668)
Supplement: Supplementary Information [file srep30668-s1.pdf]

Structural basis for misregulation of kinesin KIF21A autoinhibition by CFEOM1 disease mutations

Sarah Bianchi[#,1], Wilhelmina E. van Riel[#,2], Sebastian H. W. Kraatz[1], Natacha Olieric[1], Daniel Frey[1], Eugene A. Katrukha[2], Rolf Jaussi[1], John Missimer[1], Ilya Grigoriev[2], Vincent Olieric[3], Roger M. Benoit[1], Michel O. Steinmetz[*,1], Anna Akhmanova[*,2], Richard A. Kammerer[*,1]

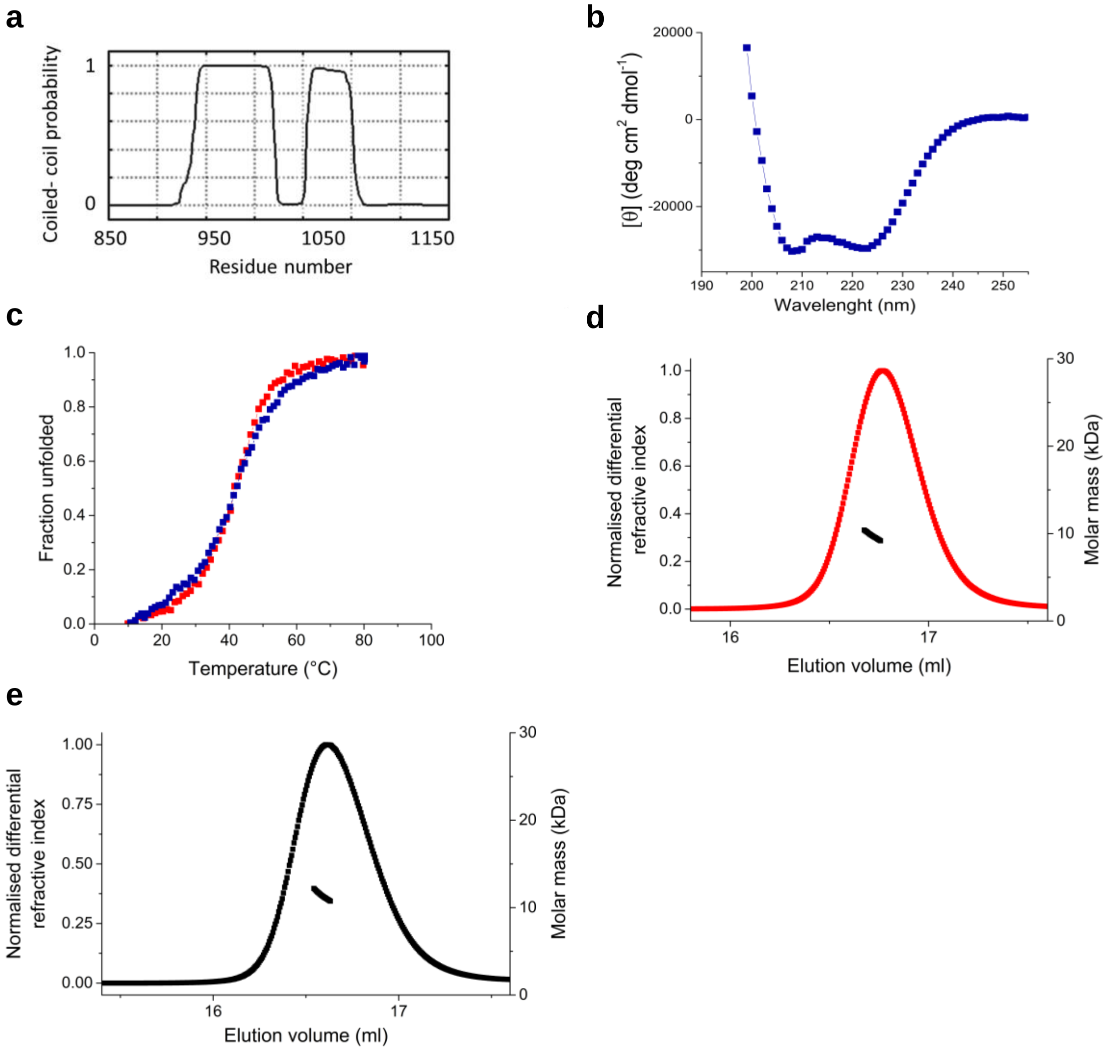

Supplementary Fig S1.

a. Coiled-coil prediction of KIF21A amino acids 850-1150 using the COILS webserver (Lupas et al, 1991).

b. Far-UV CD spectrum of rCC (blue). CD measurements were performed in PBS at 5°C using a protein concentration of 0.125mg/ml.

c. Normalised thermal unfolding profiles of rCC1 (red) and rCC (blue) recorded by CD at 222 nm. Global fitting revealed a T_m of 42.4°C for rCC1 and 42.9°C for rCC.

d. Oligomerization state of rCC1 determined by SEC-MALS at a protein concentration of 2mg/ml. The elution profile of rCC1 is visualised by the normalised differential refractive index in red and the respective experimental mass distribution in black. The theoretical mass is 9.5 kDa and the experimental mass is 10.8 kDa.

e. Oligomerization state of rCC1-L determined by SEC-MALS. The experiment was performed as described in Fig S1d. The theoretical mass is 11 kDa and the experimental mass is 11.2 kDa.

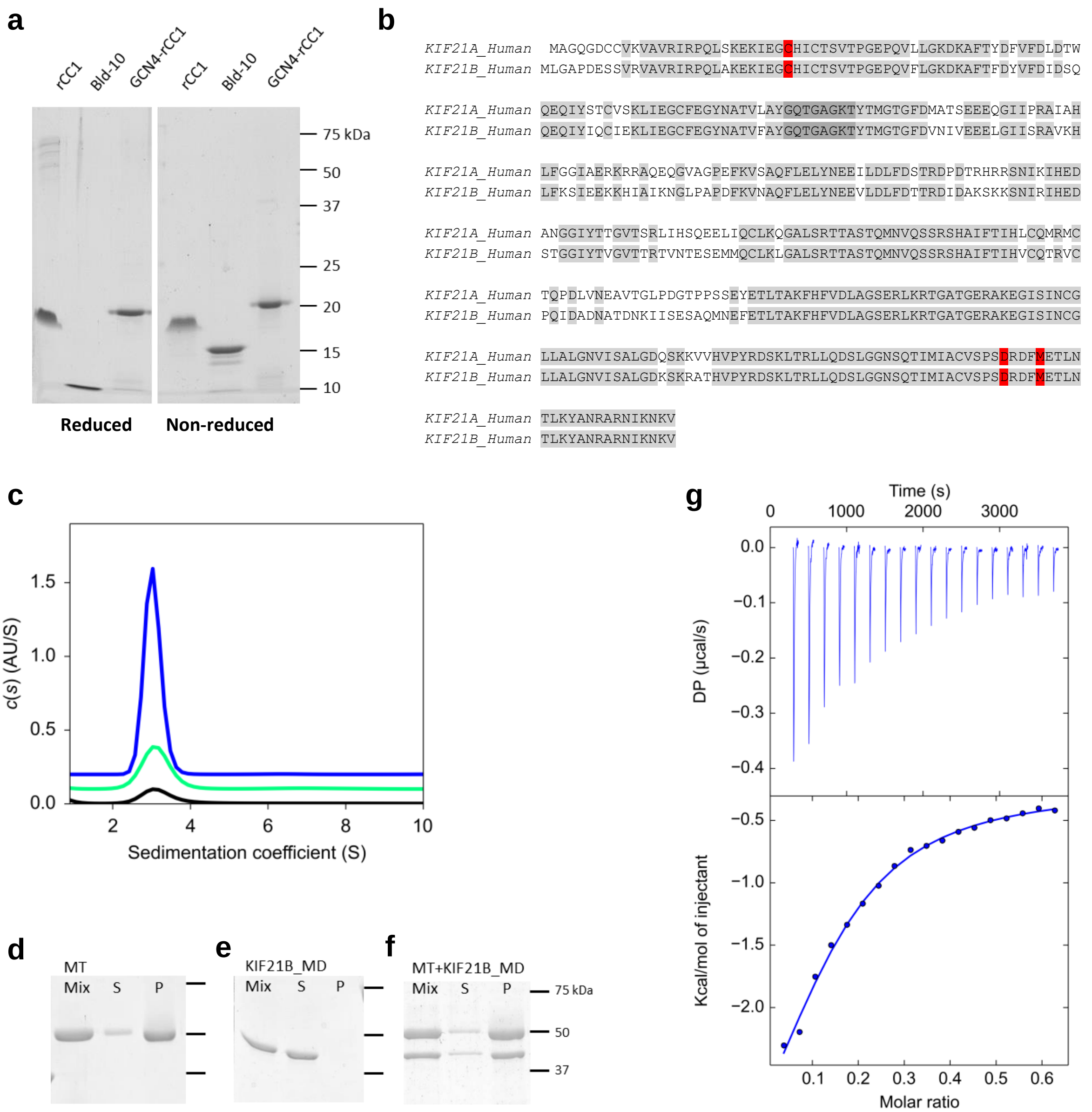

Supplementary Fig S2.

a. Cysteine crosslinking of recombinant GCN4-rCC1, rCC1 (negative control) and Bld-10 (positive control, Kraatz et al. 2016 under revision) analyzed under reducing and non-reducing conditions by SDS-PAGE.

b. Sequence alignment of *hs*KIF21A 1-375 and *hs*KIF21B 1-376. Conserved amino acids and the ATP binding region are labelled in light and dark grey, respectively. CFEOM1-associated amino acids are shown in red. The sequence identity is 75%.

c. Sedimentation velocity absorbance data using 5 (black), 15 (green) and 70 µM (blue) KIF21B_MD measured in 20 mM Tris, 150 mM NaCl and 2 mM DTT. All concentrations revealed one peak at 3.1 S only, that corresponds to a M_W of 41 kDa. This value is consistent with the theoretical M_W of a monomer (43 kDa).

d.-f. Coomassie Blue-stained SDS-PAGE gels showing mix, supernatant (S) and pellet (P) fractions of MT pelleting assays. **d** and **e** represent the controls: **d.** 2.7 µM MTs **e.** 2.8 µM KIF21B_MD. In **f.** MTs are mixed with KIF21B_MD with concentrations as in **d.** and **e.**

g. Binding affinity determination of rCC and KIF21B_MD by isothermal titration calorimetry. Experiments were performed as described in Fig 5c except that 1017µM of rCC was used in the syringe.

a

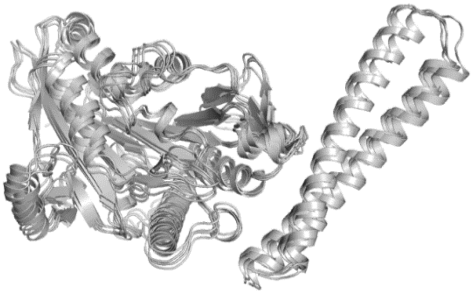

b

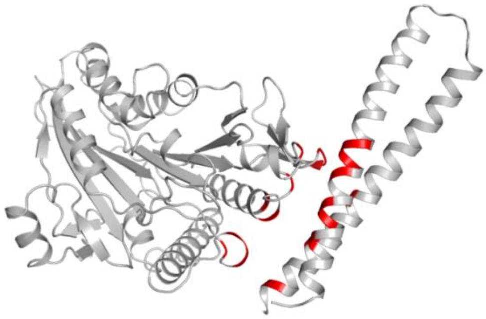

c

	M_w (theroretical)	M_w (experimental)
rCC1-L_NKQ	10.9	11.1 (0.3%)
rCC1-L_DYD	10.9	11.4 (1.4%)
rCC1-L	11.0	11.2 (2%)

d

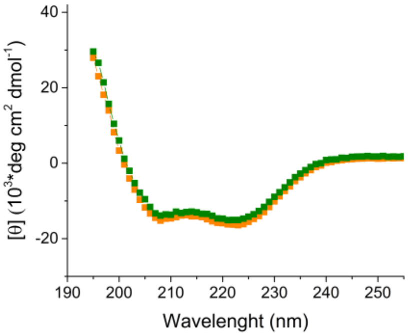

e

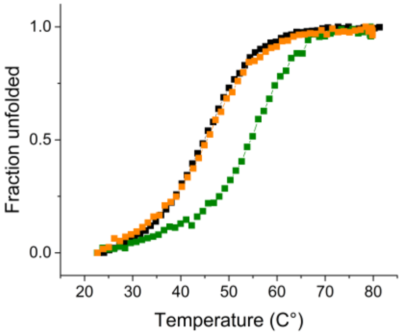

Supplementary Fig S3.

a. Superposition of the four docking models belonging to the highest-score HADDOCK cluster.

b. Docking model of KIF21A_MD and KIF21A rCC1. The interface formed between the regulatory antiparallel coiled coil and the motor domain is shown in red.

c. SEC-MALS data summary of rCC1-L WT, NKQ and DYD at 2mg/ml with the corresponding experimental M_w. Standard deviations are indicated in brackets.

d. Far-UV CD spectra of rCC1-L NKQ (orange) and DYD (green) mutants. CD measurements were performed in PBS at 5°C using a protein concentration of 12.5 μM.

e. Normalised thermal unfolding profiles of rCC1-L NKQ (orange), rCC1-L DYD (green) and rCC1-L WT (black) recorded by CD at 222 nm. Experimental conditions were as described in Figure2b and S3d.

Data collection	
Wavelength (Å)	2.06641
Resolution range (Å)	71.031 - 2.495 (2.584 - 2.495)
Space group	P 31 2 1
Unit cell abc (Å) αβγ (°)	82.02 82.02 70.57 90 90 120
Total reflections	174799 (14813)
Unique reflections	9871 (972)
Multiplicity	17.7 (15.2)
Completeness	1.00 (0.99)
Mean I/sigma(I)	15.84 (1.40)
Wilson B-factor (Å^2)	48.3
R_{merge}	0.1762 (1.785)
R_{meas}	0.1814 (1.847)
CC 1/2	0.999 (0.472)
Pearson`s CC*	1 (0.801)
Data refinement	
Reflections for R_{free}	493 (49)
Twin law	-h,-k, l
Estimated twin fraction	0.479
R_{work} / R_{free}	0.1770 (0.2598)/ 0.2034 (0.3580)
CC_{work} / CC_{free}	0.954 (0.601)/ 0.970 (0.477)
Number of non-hydrogen atoms	1331
Macromolecules	1312
Ligands	12
Water	7
Protein residues	164
RMS bonds (Å)	0.002
RMS angles (°)	0.47
Ramachandran favored (%)	98
Ramachandran allowed (%)	1.9
Ramachandran outliers (%)	0
Rotamer outliers (%)	0.68
Average B-factor (Å^2)	57.32
macromolecules	57.47
ligands	51.74
solvent	37.77

Supplementary Table S1. Crystallographic data collection and refinement statistics.
Values in parentheses correspond to the highest resolution shell.
CC1/2= percentage of correlation between intensities from random half-datasets.

	Cluster 1 (top cluster)	Cluster 4
**HADDOCK score**	-127.9 +/- 5.4	-118.5 +/- 6.5
**Cluster size**	111	36
**RMSD from the overall lowest-energy structure**	0.9 +/- 0.6	16.3 +/- 0.1
**Van der Waals energy**	-21.1 +/- 4.6	-47.7 +/- 3.0
**Electrostatic energy**	-559.3 +/- 49.5	-340.6 +/- 32.9
**Desolvation energy**	7.5 +/- 8.2	-6.5 +/- 5.3
**Restraints violation energy**	33.7 +/- 12.47	38.5 +/- 2.24
**Buried Surface Area**	1308.6 +/- 55.9	1549.2 +/- 58.3
**Z-Score**	-1.9	-1.6

Supplementary Table S2. Docking parameters derived from HADDOCK.
Values for the top two clusters derived from data-driven molecular docking using HADDOCK web server. The top cluster has a score of -122.1 (cluster 1) and the second best cluster a score of -118.5 (cluster 4).
